# Supplementary material for: Unpacking the paradoxical impact of ethical leadership on employees’ pro-social rule-breaking behavior: The interplay of employees’ psychological capital and moral identity
Source: PLoS One. 2024 Aug 22;19(8):e0306912. doi: 10.1371/journal.pone.0306912 (PMC11341026; doi:10.1371/journal.pone.0306912)
Supplement: Attachment — Submitted filename: Response to Reviewers.docx [file pone.0306912.s002.docx]

**Response to Reviewers**

I would like to extend our sincere appreciation for your prompt response and the invaluable feedback offered by the reviewers. We recognize your dedicated commitment to the review process, and we are fully devoted to addressing all comments and suggestions raised by the reviewers to enhance the overall quality of our manuscript. We have diligently attended to the queries raised by the reviewers and updated the manuscript in accordance with the recommendations provided by both the Editor and the Reviewers. Notably, the changes made to the manuscript have been highlighted in green. Additionally, we have utilized green text to draw attention to specific points raised by reviewers, where the relevant details have already been incorporated into the manuscript. We express profound gratitude to the Editor and the Reviewers for affording us the opportunity and guidance to refine the quality of our manuscript.

**Reviewer # 1**

Reviewer’s Comment

1. The introduction (Pages 6-7): I suggest writing the full terms before the abbreviations (e.g., Psychological Capital (PsyCap), Moral Identity (MID), and Social Cognitive Theory (SCT)) to improve clarity and understanding especially in the first paragraph.

Authors’ Response

We appreciate the reviewer's feedback, which has helped improve the clarity and understanding of our manuscript. Following the reviewer's suggestion, we have incorporated the full terms preceding the abbreviations in the introduction section. Subsequently, throughout the manuscript, we have employed the abbreviations as follows:

Page 3, Line 1: "Ethical Leadership (EL)"

Page 3, Line 16: "Pro-Social Rule-Breaking (PSRB)"

Page 4, Line 13: "Psychological Capital (PsyCap)"

Page 5, Line 3: "Moral Identity (MID)"

Page 5, Line 11: "Social Cognitive Theory (SCT)"

These revisions should enhance the reader's comprehension of the manuscript.

Reviewer’s Comment

2. Pro-Social Rule-Breaking Behaviour in Health Industries: Please provide a more detailed explanation of how employees in the health industry practice Pro-Social Rule-Breaking Behaviour. This will help readers better grasp the context and relevance of your study.

Authors’ Response

We appreciate the reviewer's suggestion to provide a more comprehensive explanation of how employees in the health industry, specifically nursing staff, engage in PSRB. We have addressed this by including the rationale for selecting nursing staff as the study population in the Methods Section, under "Population and Sampling," which can be found on Page 13, Lines 1 – 3. This addition provides context and relevance for our study.

Reviewer’s Comment

3. Choice of Research Design: far away of the limitations you have mentioned, Clarify the rationale behind employing a time-lagged cross-sectional design with three data collection waves (T1, T2, and T3). Why do you use this strategy which could cause CMB rather than minimize it.

Authors’ Response

We appreciate your thoughtful consideration of our research design and the raised concern about the potential common method bias (CMB) associated with our choice of a time-lagged cross-sectional design with three data collection waves (T1, T2, and T3). The rationale for this design is rooted in our aim to capture the temporal dynamics of the variables under investigation, including ethical leadership, pro-social rule-breaking, psychological capital, and moral identity. This approach aligns with recommendations by Podsakoff et al. (2012) and is supported by recent studies exploring similar topics (Dey et al., 2022; Hsieh et al., 2020; Miao et al., 2020; Park et al., 2023; Tang & Li, 2022; Wang & Shi, 2021).

We acknowledge that CMB is a potential concern in such designs. To address this, we have implemented procedural and statistical remedies, emphasizing participant confidentiality and anonymity. Additionally, unique keys/IDs were used instead of personal identifiers to minimize biases. Importantly, we collected data for different variables at distinct time points (T1, T2, and T3), strategically minimizing the likelihood of CMB by avoiding simultaneous measurement of all variables.

We hope these clarifications assure you of the validity and rigor of our study. We are grateful for your diligence in reviewing our research design and are open to further discussion or adjustments if needed.

Reviewer’s Comment

4. Data Collection Details: Provide more information about data collection from each hospital, including response rates for each, which is crucial for understanding the representativeness of your sample.

Authors’ Response

We sincerely appreciate the reviewer's valuable suggestion regarding the need for more detailed information about data collection from each hospital, including response rates for each. In response to this insightful feedback, we have included a comprehensive table, Hospital Wise Response Rate (Table 1), on Pages 15 - 16. This addition aims to provide a clearer understanding of the representativeness of our sample, and we trust that it enhances the overall robustness of our study.

Reviewer’s Comment

5. You mentioned conducting a Common Method Bias (CMB) test, please included the results in the paper for transparency and to support your research validity.

Authors’ Response

We appreciate the reviewer's suggestion to include the results of the Common Method Bias (CMB) test in the paper for transparency and to support research validity. In response to this, we have now included the results of The Harman Single-Factor test in the Methods Section under Data Analysis on Page 20, Lines 8 - 11. We hope that this addition enhances the transparency and validity of our research.

Reviewer’s Comment

6. Factor Loadings (Items EL7, PC11, PC12) are below 0.70. Explain your reasoning for retaining these items in the analysis, as they could impact the reliability and validity of your results.

Authors’ Response

Thank you for your valuable feedback regarding the factor loadings of items EL7, PC11, and PC12. We acknowledge your concern and appreciate the opportunity to clarify our rationale for retaining these items in the analysis.

While it's true that these specific items have factor loadings below the recommended threshold of 0.70, they still demonstrate acceptable loadings above 0.60. Importantly, other reliability and validity measures, including Chronbach’s alpha (α), composite reliability (CR), and average variance extract (AVE), support the overall robustness of our measurement model. All study constructs exhibit good internal consistency (α and CR > 0.708) and convergent validity (AVE > 0.50) (Becker et al., 2023; Hair et al., 2019).

We have included this information in the Results Section under the Measurement Model on Page 22, Lines 12 – 17, providing transparency and justification for our decision to retain these items. We believe that these findings collectively contribute to the reliability and validity of our results.

Your insights are invaluable, and we hope this clarification addresses your concerns. We remain open to further discussion and adjustments as needed.

Reviewer’s Comment

7. Include the results of Fornell-Larcker criteria in your paper.

Authors’ Response

Thank you for your insightful suggestion regarding the inclusion of Fornell-Larcker criteria results in our paper to assess discriminant validity. We appreciate your guidance, and we have now incorporated the Fornell-Larcker criteria results into our manuscript as Table 7 in the Results Section under the Measurement Model on Page 25.

These additional results offer further evidence of the discriminant validity of our study constructs. We trust that this addition enhances the clarity and rigor of our paper. Your feedback has been instrumental in refining our work, and we remain open to any further recommendations you may have.

Reviewer’s Comment

8. In page 23, you mentioned VIF values, but it appears you are referring to variables rather than indicators. Please include the VIF values for the indicators to clarify this point.

Authors’ Response

Thank you for your astute observation and valuable feedback regarding our reference to VIF values for variables rather than indicators. We appreciate your diligence, and to address this concern, we have included the Variance Inflation Factor (VIF) values specifically for the indicators measuring the outer model in our manuscript. These values are now presented in Table 6 on Pages 22 - 24.

We believe that this addition clarifies the point you raised and contributes to the overall transparency and precision of our paper.

Reviewer’s Comment

9. F2 values is 0.038 which indicate to small effect size and not large effect size. Moreover, Q2 is 0.242 which is weak and not large. Ensure the interpretations accurately reflect the analysis outputs.

Authors’ Response

We sincerely appreciate your careful review and feedback on the interpretation of the F2 and Q2 values in our manuscript. Your observation regarding the size of the effect and the strength of predictive accuracy is valuable, and we have thoroughly reviewed and revised our interpretations to accurately reflect the analysis outputs.

The corrected interpretations can now be found in the Results Section under the Structural Model at Page 27, Lines 17 - 18, and Page 28, Lines 1 - 3. We have acknowledged that the F2 value of 0.038 indicates a small effect size, and the Q2 value of 0.242 is considered moderate rather than large.

Thank you for ensuring the precision and clarity of our analysis interpretations. Your diligence has contributed to the rigor of our study.

References

Becker, J. M., Cheah, J. H., Gholamzade, R., Ringle, C. M., & Sarstedt, M. (2023). PLS-SEM’s most wanted guidance. International Journal of Contemporary Hospitality Management, 35(1), 321-346.

Dey, M., Bhattacharjee, S., Mahmood, M., Uddin, M. A., & Biswas, S. R. (2022). Ethical leadership for better sustainable performance: Role of employee values, behavior and ethical climate. Journal of Cleaner Production, 337, 130527.

Hair, J. F., Risher, J. J., Sarstedt, M., & Ringle, C. M. (2019). When to use and how to report the results of PLS-SEM. European business review, 31(1), 2-24.

Hsieh, H. H., Hsu, H. H., Kao, K. Y., & Wang, C. C. (2020). Ethical leadership and employee unethical pro-organizational behavior: a moderated mediation model of moral disengagement and coworker ethical behavior. Leadership & Organization Development Journal, 41(6), 799-812.

Miao, Q., Eva, N., Newman, A., Nielsen, I., & Herbert, K. (2020). Ethical leadership and unethical pro‐organisational behaviour: The mediating mechanism of reflective moral attentiveness. *Applied Psychology*, *69*(3), 834-853.

Park, J. G., Zhu, W., Kwon, B., & Bang, H. (2023). Ethical leadership and follower unethical pro-organizational behavior: examining the dual influence mechanisms of affective and continuance commitments. The International Journal of Human Resource Management, 1-31.

Podsakoff, P. M., MacKenzie, S. B., & Podsakoff, N. P. (2012). Sources of method bias in social science research and recommendations on how to control it. Annual review of psychology, 63, 539-569.

Tang, Y., & Li, Y. (2022). Ethicality neutralization and amplification: a multilevel study of ethical leadership and unethical pro-organizational behavior. *Journal of Managerial Psychology*, *37*(2), 111-124.

Wang, F., & Shi, W. (2021). Inclusive leadership and pro-social rule breaking: the role of psychological safety, leadership identification and leader-member exchange. Psychological Reports, 124(5), 2155-2179.

**Reviewer #2**

Reviewer’s Comment

Abstract

It might be beneficial to finish the abstract by discussing the larger significance of these results for organizational leadership and suggesting future research alternatives in this field. This would bring the narrative to a close and offer the reader with a clear takeaway message.

Authors’ Response

We express our gratitude for your insightful suggestion to enhance the conclusion of the abstract by discussing the broader significance of our results for organizational leadership and proposing future research directions in the field. Your input has been invaluable, and we have promptly incorporated this recommendation into our manuscript.

The revised abstract, now concluding with a discussion on the larger significance of our results and suggestions for future research, can be found on Page 2 from Lines 12 - 16. We trust that this addition brings a more comprehensive and conclusive perspective to our abstract, offering readers a clear takeaway message.

Thank you for your constructive feedback, which has undoubtedly strengthened the quality and relevance of our study.

Reviewer’s Comment

Introduction

In general, the introduction has useful information, but it might be improved by simplifying, giving precise terminology (e.g., PsyCap, MID), and specifying particular study aims or questions. This will provide the study with a more focused and ordered foundation.

Authors’ Response

We appreciate your thoughtful feedback on the introduction section of our manuscript. Your suggestion to simplify the introduction, provide precise terminology, and specify particular study aims or questions is well-received.

In response to your valuable input, we have revised the introduction, simplifying the language and incorporating precise terminology. We provided precise terminologies with full terms preceding the abbreviations appearing for the first time in the text at Page 3, Lines 1 and 16, Page 4, Line 13 and Page 5 Line 3 and 11. Additionally, we have specified the particular study aims and questions, making the foundation more focused and ordered on Pages 5 - 6.

We trust that these revisions contribute to the improved clarity and organization of our study, aligning with your recommendations. Thank you for guiding us in enhancing the quality of our manuscript.

Reviewer’s Comment

Theory and Hypotheses Development

1. Rather than using the word "paradoxical relationship" without explanation, the text should explain why the observed positive association between ethical leadership and PSRB is regarded as paradoxical.
2. The statement "shedding light on the dynamic nature of ethical leadership" is a little ambiguous. A more specific explanation of how the findings contribute to our knowledge of ethical leadership dynamics would be helpful in this area.
3. It is critical to address any potential critiques or limitations connected with the selected theoretical framework when introducing it. This indicates a sophisticated comprehension of the theoretical perspective.

Authors’ Response

We want to express our sincere appreciation for your valuable feedback on our manuscript, particularly regarding the Theory and Hypotheses Development section. Your insights have proven instrumental in refining our theoretical framework and enhancing the overall clarity of our research.

In response to your suggestions, we have provided a more comprehensive explanation of why the observed positive association between ethical leadership and pro-social rule-breaking behavior (PSRB) is considered paradoxical. This clarification is now integrated into the revised literature review, offering readers a deeper understanding of the theoretical underpinnings.

Furthermore, we have addressed the ambiguity in the statement about "shedding light on the dynamic nature of ethical leadership." The revised version now provides a specific explanation of how our findings contribute to advancing knowledge about the complex and multifaceted dynamics of ethical leadership.

Finally, we have taken your advice to heart and diligently addressed potential critiques and limitations associated with our chosen theoretical framework. This revised literature review reflects a more sophisticated grasp of the theoretical perspective, ensuring a more robust foundation for our research.

We genuinely believe that these revisions have significantly improved the quality and coherence of our manuscript. We are grateful for your thorough review and constructive comments, which have undoubtedly strengthened our work.

Methods

Population and Sampling

Reviewer’s Comment

1. Rationale for Sample Size: Explain why a higher sample size (500) was chosen, even when the GPower calculator indicated a minimum of 150. The methodological basis would be better if the reasons behind this decision were clarified.

Authors’ Response

We appreciate your insightful feedback regarding our rationale for choosing a sample size of 500, especially when the GPower calculator suggested a minimum of 150. Your comment prompted us to provide a more thorough explanation of the methodological basis behind this decision.

In response, we have clarified in the manuscript's Methods Section under Sample Size (Page 13, Lines 12 – 17) why a higher sample size was deemed necessary. We emphasized that while the GPower calculator's minimum is suitable for basic statistical analyses, the complexity of multivariate statistical methods like Partial Least Squares Structural Equation Modeling (PLS-SEM) warrants larger sample sizes. Citing scholarly recommendations, such as Jobst, Bader, & Moshagen (2023), Lakens (2022), and Mumtaz et al. (2020), along with the guidance of Comrey & Lee (2013), who consider 500 as a very good size for PLS-SEM analyses, we aimed to provide a robust methodological foundation for our chosen sample size.

We believe this clarification addresses your concerns and enhances the transparency of our research approach. Thank you for guiding us toward a more comprehensive explanation.

Reviewer’s Comment

2. Data Collection Procedure: Include a section explaining the quality control procedures used during data collection, including the efforts required in order to ensure accurate and reliable responses.

Authors’ Response

We express our gratitude for your insightful comment, prompting us to include a section elucidating the quality control procedures employed during data collection. Recognizing the paramount importance of ensuring accuracy and reliability in scientific research, we have already incorporated a comprehensive explanation of our quality control measures in the Methods Section (Pages 13-15).

Our implemented measures, such as maintaining survey instrument consistency, including a cover letter for confidentiality and voluntary participation, encouraging anonymous responses, adopting a time-lagged cross-sectional research design, employing systematic coding, and providing clear definitions, collectively form a robust framework for enhancing the reliability and accuracy of our collected data.

We trust that this explanation strengthens the transparency and rigor of our data collection procedures. Your feedback has been invaluable in refining our manuscript, and we appreciate the opportunity to address this crucial aspect of our research.

Reviewer’s Comment

3. Measurement Validity and Reliability: Add a paragraph outlining the measurement instrument's reliability and validity, as well as references to any relevant validation studies.

Authors’ Response

Thank you for your valuable feedback. We wish to bring to your attention that we have already addressed the reliability and validity of the measurement instruments in our manuscript. The relevant information is presented in the 'Measurements' section on Pages 19 - 20, where we have outlined the properties of the measurement instruments, including details about their reliability and validity. Additionally, we have referenced relevant validation studies to support the credibility of our measurement tools.

We hope this clarification helps, and we encourage you to review the specified section for a thorough understanding of the measurement instrument's reliability and validity. Your meticulous review has significantly contributed to the enhancement of our manuscript, and we appreciate your continued engagement.

Reviewer’s Comment

4. Ethical Considerations: Since you stated getting permission from the hospitals, consider including a statement about ethical approval and any actions taken to protect the confidentiality of participants and informed consent. Include a section addressing the study's ethical issues, such as permission from appropriate ethics boards and safeguards in place to preserve the confidentiality of participants and rights.

Authors’ Response

We greatly appreciate your insightful feedback, and we are pleased to inform you that we have incorporated your suggestions into our manuscript. Specifically, we have included a dedicated section titled "Ethical Considerations" on Pages 20 - 21 to address the ethical aspects of our study comprehensively. In this section, we provide a detailed account of the ethical principles guiding our research, emphasizing the steps taken to secure authorization from the relevant hospitals, protect participant confidentiality, and ensure informed consent. Furthermore, we explicitly state that our research received ethical approval from the university review board, affirming our adherence to international ethical standards.

We trust that this addition strengthens the ethical framework of our study, and we are grateful for your guidance in enhancing the transparency and rigor of our research.

Results

Reviewer’s Comment

1. Structural Model Evaluation:

Provide a brief analysis of the R2, F2, and Q2 values, emphasizing their significance for the model's explanatory and predictive abilities.

Authors’ Response

We sincerely appreciate your feedback and have incorporated a brief analysis of the R^2^, F^2^, and Q^2^ values into our manuscript. This analysis is presented in the Results Section under the Structural Model on Pages 27 - 28, emphasizing the significance of these values for evaluating the model's explanatory and predictive abilities.

We trust that this addition enhances the clarity and interpretability of our results, and we are grateful for your valuable input.

Discussion

Reviewer’s Comment

1. Interpretation of Contradictory Results: Are there contextual factors or specific organizational dynamics that might contribute to this unexpected outcome?

Authors’ Response

We appreciate your attention to the unexpected outcome and have addressed this concern in the Discussion Section on Page 31, Lines 15 - 22. We thoroughly explored contextual factors and organizational dynamics that might contribute to this result. We hope that our discussion provides a comprehensive explanation for the observed outcome. Thank you for bringing this to our attention.

Reviewer’s Comment

2. Interpretation of the Counterintuitive Finding:

Authors’ Response

Thank you for highlighting the counterintuitive finding. We have provided an interpretation and discussion of this unexpected result in the Discussion Section on Page 32, Lines 1 - 10. We hope this explanation adequately addresses your inquiry.

Reviewer’s Comment

3. Implications for EL Perception: Could this finding imply that workers understand ethical leadership differently than previously thought?

Authors’ Response

Thank you for your insightful question regarding the implications for ethical leadership perception. We have discussed this aspect in the Discussion Section on Page 32, Lines 1 - 10, where we explain the counterintuitive finding. We believe that this explanation adequately addresses your concern.

Reviewer’s Comment

4. Distinguishing Pro-Social Intentions:

• Distinguishing Intentions from Actions: Add a brief remark to highlight that the research is concentrating on pro-social intentions and behaviors, even if they require breaking specific norms.

Authors’ Response

Thank you for your valuable suggestion to distinguish intentions from actions. We have incorporated a brief remark in the Discussion Section on Page 32, Lines 6 - 10, emphasizing our research focus on pro-social intentions and behaviors, even if they involve breaking specific norms. We believe this addition enhances the clarity of our research perspective.

Reviewer’s Comments

5. Mediation Role of PsyCap:

• Potential Mechanisms: Investigate potential mechanisms or specific components of PsyCap that may be particularly relevant in mediating the link between EL and PSRB.

Authors’ Response

Thank you for your valuable suggestion to explore potential mechanisms within PsyCap. In response, we have included a future research direction in the Discussion Section, specifically under Limitations and Future Research Directions on Page 38, Lines 11 - 14. This highlights the need for further investigation into specific components or mechanisms within PsyCap that may play a pivotal role in mediating the link between EL and PSRB. We believe this addition aligns with your suggestion and enhances the scope for future research in this domain.

Reviewer’s Comments

6. Discussion of Moderating Effect:

• Further Implications of MID: How might individuals with higher MID levels contribute differently to the overall organizational climate?

Authors’ Response

Thank you for your insightful inquiry into the further implications of moral identity (MID). In response, we have integrated future research directions into the Discussion Section under Limitations and Future Research Directions on Page 38, Lines 15 - 18, addressing how individuals with higher MID levels might contribute differently to the overall organizational climate. Additionally, we expanded our future research directions to encompass different antecedents of PSRB, such as authentic and servant leadership, as indicated on Page 39, Lines 1 - 5. Furthermore, we included considerations of different consequences of ethical leadership, including Unethical Pro-Organizational Behavior (UPB) and whistleblowing, as highlighted on Page 38, Lines 19 - 21. To enhance the model's predictive relevance, we suggested exploring additional factors contributing to PSRB, as outlined on Page 39, Lines 6 - 8. We believe these additions strengthen the potential avenues for future research in this domain.

**Reviewer #3**

Reviewer’s Comment

1. The topic is quite interesting, but I am sharing a few observations which may be incorporated in the next version of the manuscript.

Authors’ Response

Thank you for your feedback, and we appreciate your positive remarks about finding our topic interesting. We are committed to incorporating your valuable observations into the next version of the manuscript to improve its overall quality. Your guidance is highly appreciated.

Reviewer’s Comment

2. Why the use of SPSS? Please clarify because descriptive statistics can also be done through PLS-SEM.

Authors’ Response

Thank you for your inquiry. We used SPSS for data entry, coding, and editing due to its widespread use and familiarity in the initial stages of data preparation. While PLS-SEM software can compute descriptive statistics, SPSS was chosen for its extensive and user-friendly features for these tasks. This aligns with common research practices where SPSS is employed for preliminary data assessments before advanced analyses with PLS-SEM (Abbas et al., 2022; Park et al., 2023; Tang & Li, 2022). We appreciate your understanding and hope this clarification addresses your concern.

Reviewer’s Comment

3. Do mention the original scale of the following construct EL, MID, PC, PSRB

Authors’ Response

Thank you for your comment. The original scales of the constructs EL, MID, PC, and PSRB have been clearly outlined in the Methods Section under Measurement on Pages 18 - 20. We appreciate your attention to detail, and we hope that this information is sufficient for readers to grasp the measurement instruments used in our study.

Reviewer’s Comment

4. Specify Outer VIF Values and Lacker criteria

Authors’ Response

We appreciate the reviewer's inquiry regarding the Outer VIF Values and Fornell-Larcker Criterion. The Outer VIF Values are specified in Table 6 on Pages 22 - 24, demonstrating that all indicators have VIF values below 3.0, indicating the absence of collinearity. Additionally, the Fornell-Larcker Criterion results are presented in Table 7 on Page 25, where the AVE of all constructs exceeds their respective correlations with other study constructs, confirming discriminant validity. We hope this information assures you of the robustness of our measurement model.

Reviewer’s Comment

5. Please recheck H1 EL > PSRB P value is 0.002, but the author mentioned it is not supported, which is incorrect.

Authors’ Response

Thank you for bringing this to our attention. We acknowledge the need for clarification regarding H1. While the p-value of 0.002 indicates statistical significance, our original hypothesis, anticipating a negative relationship between ethical leadership and employees' pro-social rule-breaking, was not supported in the expected direction. We have clarified this in the Results Section under Hypotheses Testing on Page 29, Lines 5 - 7. We appreciate your diligence in ensuring accuracy and value your input, which has contributed to improving the precision and clarity of our presentation.

Reviewer’s Comment

6. Page no 21 Table 3: Items under EL (10), MID (05), PC (12), PSRB (13), but in Figure 2, Items are mismatched.

Authors’ Response

Thank you for your careful review, and we sincerely appreciate your diligence in identifying the discrepancy between the items listed under each construct in Table 3 and their representation in Figure 2. We have taken immediate action to rectify this issue. The Research Model has been re-run in Smart PLS, and a new Figure 2 has been generated to ensure that all items now align correctly with the construct they belong to. The updated Figure 2 on Page 21, and the corrected Table 6 on Pages 22 - 24, have now been included in the revised manuscript in the Results Section under the Measurement Model. We trust that these revisions have resolved the mismatch, and we thank you for bringing this to our attention.

Reviewer’s Comment

7. You may please refer to the following relevant references for your further perusal:

https://doi.org/10.1108/QRFM-04-2017-0028

https://doi.org/10.1108/QRFM-07-2018-0081

https://doi.org/10.1016/j.jclepro.2023.138441

<https://doi.org/10.1111/ijcs.12850>

Authors’ Response

Thank you for providing additional references for our consideration. We have thoroughly examined the provided references and, while they are valuable contributions to the field, we have determined that they do not directly align with the focus and scope of our current manuscript. We appreciate your efforts to enrich our research, and we will certainly consider relevant references in our future work.

References

Abbas, A., Saud, M., Suhariadi, F., Usman, I., & Ekowati, D. (2022). Positive leadership psychology: Authentic and servant leadership in higher education in Pakistan. Current Psychology, 41(9), 5859-5871.

Park, J. G., Zhu, W., Kwon, B., & Bang, H. (2023). Ethical leadership and follower unethical pro-organizational behavior: examining the dual influence mechanisms of affective and continuance commitments. The International Journal of Human Resource Management, 1-31.

Tang, Y., & Li, Y. (2022). Ethicality neutralization and amplification: a multilevel study of ethical leadership and unethical pro-organizational behavior. Journal of Managerial Psychology, 37(2), 111-124.

**Reviewer #4**

Reviewer’s Comment

1. The article is about ethical leadership and found to be good. The article needs to be strengthened and introduced well on various aspects of ethical leadership.

Authors’ Response

We appreciate your positive feedback on the article and acknowledge your suggestion to strengthen and introduce various aspects of ethical leadership in the introduction. In response to your valuable input, we have revisited and revised the introduction to enhance its content and provide a more comprehensive overview of the different facets of ethical leadership. We believe that these revisions will contribute to a more robust and well-introduced manuscript. Thank you for your guidance.

Reviewer’s Comment

2. The introduction needs to be focused on relevant literature, currently it seems to verbose and difficult to follow.

Authors’ Response

We sincerely appreciate your positive feedback and are thankful for your constructive suggestions to improve the focus and clarity of the introduction. We have carefully revisited and revised the Introduction Section to ensure it is more focused on relevant literature and easier to follow. We believe these changes contribute to a more cohesive and streamlined introduction that aligns with the core themes of our research.

Reviewer’s Comment

3. The sampling have to be explained and justified in a manner it could be replicated in other places and defined well.

Authors’ Response

We appreciate your insightful feedback on our manuscript, particularly regarding the clarity and replicability of our sampling procedure. Your comments have been instrumental in refining our work. In response to your suggestion, we have made comprehensive revisions in the Methods Section under Population and Sampling (Pages 12 – 13), clarifying the sampling technique (Page 13, Lines 6 – 9) and justifying the chosen sample size (Page 13, Lines 14 - 19). Additionally, we have highlighted the data collection procedures on Pages 14 – 15. These modifications are aimed at enhancing the transparency and replicability of our sampling approach for future research. We sincerely appreciate your guidance and the opportunity to improve our manuscript.

Reviewer’s Comment

4. The article provide results in table but not highlighted the relevant results having policy implication in the text.

Authors’ Response

We sincerely appreciate your insightful feedback regarding the need to highlight results with policy implications in the text. Your suggestion has been invaluable in refining our manuscript. In response, we have revisited and revised the Discussion Section, providing a detailed discussion of the policy implications of our results on Pages 30 - 34, as well as managerial implications on Pages 36 - 37. We believe that this revision enhances the practical relevance of our study and aligns with your expectations.

Reviewer’s Comment

5. The discussion section needs to be improved by including studies of developing countries instead of skewed representation of work.

Authors’ Response

We sincerely appreciate your thoughtful suggestion to improve the discussion section by incorporating studies from developing countries. Your input has been invaluable in refining our manuscript. In response, we have revisited and revised the Discussion Section, now including studies from developing countries such as Iran, Iraq, Turkey, and Pakistan, to provide a more balanced and inclusive representation of the relevant literature (Al Halbusi et al., 2023; Bouckenooghe et al., 2015; Bouzari et al., 2020; Tufan et al., 2023; Yasir & Rasli, 2018) on Page 30, Lines 15 - 17. We believe that this update enhances the comprehensiveness of our discussion and aligns with your suggestion.

Reviewer’s Comment

6. The study needs a focused and actionable conclusion on ethical leadership by interpreting the data and results.

Authors’ Response

We sincerely appreciate your constructive feedback, which has been instrumental in improving the quality of our manuscript. In response to your insightful input, we have diligently included the Conclusion section on Page 39. We emphasize the need to reassess traditional notions of ethical leadership in light of our data and results, taking into account the influence of contextual factors. Our conclusion underscores the importance of tailored leadership training, the significance of nurturing employees' psychological capital, and the motivation derived from individuals with strong moral identities. We also highlight the crucial role of aligning ethical leadership with contextual needs to cultivate an ethical workplace culture. These refinements aim to provide a more impactful and actionable conclusion, in line with your suggestion. We are grateful for your guidance, which has contributed to enhancing the overall effectiveness of our manuscript.

Reviewer’s Comment

7. Overall, the study is conveying a message and need to be improved.

Authors’ Response

We genuinely appreciate your acknowledgment of our study and the constructive feedback you provided. In response to your thoughtful suggestions, we have diligently revisited and revised various sections of our manuscript to address your concerns and improve its overall quality. We hope that these revisions align with your expectations and enhance the clarity and impact of our work. We are thankful for your valuable guidance, which has undoubtedly contributed to the refinement of our manuscript.

**Reviewer #5**

Reviewer’s Comment

1. In the introduction, please discuss your gap more prominently. It is suggested to provide strong logic of the mediating and moderating inclusions instead of claiming that this study is the first to test these relationships.

Authors’ Response

We sincerely appreciate your insightful feedback on our manuscript. In response to your suggestion, we have revisited and revised the Introduction section to more prominently discuss the research gaps and provide a stronger rationale for the inclusion of mediation and moderation in our study. We believe these changes enhance the clarity and depth of our introduction, and we are thankful for your guidance in improving the overall quality of our manuscript.

Reviewer’s Comment

2. For the methods sections-The data was collected using English as the medium of instruction. My very basic concern is that most of the nurses are not well-educated in Pakistan. How come the provided questionnaire can yield a very productive and non-biased outcome?

Authors’ Response

We genuinely appreciate your thoughtful consideration and concern regarding the language of data collection in our study. We would like to highlight that the nursing education system in Pakistan is well-established, with a significant number of registered nurses and recognized institutions offering nursing diploma and degree programs. Our study specifically focused on nursing staff with at least a bachelor's degree, meeting the educational qualifications set by the Pakistan Nursing Council (PNC).

In Pakistan, the use of English as the medium of instruction in colleges, universities, and organizational settings is widespread, and nursing education is no exception. To address language-related concerns, we took several measures, including attaching a letter to the questionnaires explaining the study's purpose and ensuring confidentiality. Moreover, respondents were not required to provide personal information, and operational definitions were provided for clarity.

The distribution of questionnaires in English aligns with prevalent research practices in management sciences in Pakistan, as evident in prior studies (Abbas et al., 2014; Abbas & Raja, 2015; Haq et al., 2022; Murtaza et al., 2016). We believe that these considerations, combined with the targeted educational qualifications, effectively address and mitigate potential biases related to language proficiency among the surveyed nursing staff.

We hope this clarification addresses your concerns, and we appreciate the opportunity to provide further insights into our methodology.

Reviewer’s Comment

3. Along with that, the key concepts should be defined and operationalized from the start. Instead of providing your definitions in the hypotheses provide them in the introduction for more convincing flow of this section.

Authors’ Response

We appreciate your insightful feedback and the opportunity to improve the coherence and accessibility of our manuscript. In response to your valuable suggestion, we have restructured the manuscript by moving the definitions of key constructs from the Literature Review section to the Introduction section. The revised definitions are now presented in the Introduction on Page 3, Lines 2 – 4, Lines 16 – 18; Page 4, Lines 15 – 21, and Page 5, Lines 5 - 6. We believe this modification aligns with your recommendation and contributes to a more convincing flow of the manuscript. Thank you for guiding us in enhancing the clarity and organization of our paper.

Reviewer’s Comment

4. Second, the authors have provided a statement with university degrees. Okay we for one moment take this point in consideration as valid. My again a very basic question is that how many nurses are well-educated with nursing degrees?

Authors’ Response

We appreciate your insightful comment and acknowledge the importance of considering the educational qualifications of nurses in our study. In response to your concern, we have gathered specific data from the Pakistan Nursing Council (PNC) to provide clarity on the educational background of registered nurses in Pakistan. According to the PNC, out of the total 108,396 registered nurses, 20,413 hold a Bachelors in Nursing, 574 have a Masters in Public Health, 722 possess an MS in Nursing, and 11 have a Ph.D. in Nursing. We hope this information addresses your concern, and we appreciate your diligence in ensuring the robustness and relevance of our study.

Reviewer’s Comment

5. Third, the authors have stated that they have collected their data in 3 waves with 751 in the first round, 649 questionnaires in the second round and finally 551 nurses in the third round. How the authors have ensured that nurses have university degrees. Where most of the nurses are not university qualified.

Authors’ Response

Thank you for your insightful comments and concerns. We appreciate the opportunity to address the issue raised regarding the educational qualifications of nurses in our study. To address this concern, we already highlighted that out of 108,396 registered nurses, 20,413 hold a Bachelors in Nursing, 574 have a Masters in Public Health, 722 possess an MS in Nursing, and 11 have a Ph.D. in Nursing.

To ensure the reliability and validity of our findings, we specifically targeted nursing staff with at least a bachelor's degree, consistent with the requirements set by the Pakistan Nursing Council (PNC). This criterion was clearly communicated to the hospital management, who played a pivotal role in identifying and reaching out to eligible nursing staff.

Furthermore, we took additional steps to verify the educational qualifications of participants. We directly contacted hospital management, provided them with a detailed overview of the study's objectives, and sought their collaboration in obtaining a list of registered nursing staff with a minimum bachelor's qualification. This list served as the basis for randomly selecting willing participants, thus reinforcing the inclusion of qualified individuals in our study.

In our questionnaire design, we included options for participants to indicate their education qualifications, offering an additional layer of assurance that individuals without at least a bachelor's degree were not considered for inclusion. The combination of these measures aimed to address concerns about the educational background of our participants and ensure the robustness of our data.

We hope this clarification adequately addresses your concerns and improves the quality of our manuscript.

Reviewer’s Comment

6. What was the sampling technique which is employed. Was it probability or non-probability? There is no information in this regard. Please provide more explanation in this regard.

Authors’ Response

Thank you for your insightful comment regarding the clarification of our sampling technique. We acknowledge the importance of providing a thorough explanation of the methodology employed in our study. In response to your suggestion, we have explicitly stated in the manuscript that we utilized a purposive sampling technique, which falls under the category of non-probability sampling methods. This choice was driven by practical constraints related to the unavailability of a comprehensive sampling frame covering all public and private hospitals in our target cities.

To ensure clarity and transparency, we have incorporated this information into the Methods Section under Population and Sampling on Page 13, Lines 6 - 9. We believe that this addition enhances the understanding of our sampling methodology and provides the necessary context for evaluating the generalizability of our findings.

Reviewer’s Comment

7. Your demographics states that around 230 nurses have 1-5 yrs of experience. Now how we can ensure that they have less than 3 or more than 3 yrs of experience. Less experience means they are not very well familiar with the overall management issues and ethical leadership concerns. This point is very serious.

- Therefore, please provide a very strong justification in this regard. This point is in turn making your chances of publication very low.

Authors’ Response

We appreciate your thoughtful consideration of our study and your specific concern about the categorization of nursing staff based on years of experience. Your feedback has prompted us to revisit and reevaluate our approach to presenting demographic information.

In response to your concerns, we want to clarify that the intention behind creating the 1-5 years bracket was not to establish a strict differentiation between nurses with less than 3 years and those with more than 3 years of experience. Instead, the goal was to encompass various stages in nursing careers and provide a broad overview of the distribution of experience levels within our sample.

We acknowledge the potential variations within the 1-5 years category and understand that the familiarity with overall management and ethical leadership issues may differ among individuals within this bracket. However, this logic would also equally apply for the respondents who fall within the 3-5 years range

Moreover, even if we consider the 230 respondents within the 1-5 years bracket as not very familiar, we still have 285 respondents (55.3% of our sample) who are sufficiently to excellently familiar with overall management and ethical leadership issues. This far exceeds the minimum requirement of 150 sample size suggested by the G Power formula for this study.

Furthermore, we would like to emphasize that our decision to categorize employees into the 1-5 years bracket is consistent with common practices in management sciences, as demonstrated in recent studies (Al Halbusi et al., 2023; Haq et al., 2022; Kuenzi et al., 2020; Tang & Li, 2022). Additionally, we highlight a recent study published in the Journal of Cleaner Production, which categorized respondents into brackets of ten years each, including a bracket of 1–10 years, further supporting our practice in the field.

We hope this clarification addresses your concerns and assures you of the validity and appropriateness of our approach to presenting demographic information. We appreciate your diligence in reviewing our manuscript.

Reviewer’s Comment

8. Please follow the standard format of R2 Q2 and F2 instead of R2, Q2 and F2.

Authors’ Response

Thank you for your attention to detail and your suggestion to follow the standard format of R^2^, Q^2^ and F^2^. We acknowledge and appreciate your input. In response to your comment, we have made the necessary corrections throughout the Results and Discussion Sections, consistently adopting the standard format of R^2^, Q^2^ and F^2^ wherever these terms appear in the text. We believe that this adjustment aligns with the standard format and enhances the overall consistency and professionalism of our manuscript.

We value your feedback and are committed to ensuring the quality and clarity of our manuscript. Thank you for your time and consideration.

Reviewer’s Comment

9. Please provide more justifications to strengthen your managerial implications. Provide some actionable suggestions based on your arguments to enhance the clarity of this section.

Authors’ Response

We sincerely appreciate your feedback and your insightful suggestion to provide more justifications and actionable suggestions to strengthen the managerial implications in our manuscript. In response to your valuable input, we have revisited and revised the Managerial Implications Section on Pages 36 – 37.

We have worked to provide additional justifications for our managerial implications, tying them more explicitly to our study's findings and the broader context. Moreover, we have included actionable suggestions based on our arguments to enhance the clarity and practical value of this section.

We trust that these revisions effectively address your concerns and contribute to the overall improvement of our manuscript.

Reviewer’s Comment

10. Please proofread your document as there are several grammatical errors that are breaking the smooth flow of reading.

Authors’ Response

We are genuinely grateful for your feedback regarding the grammatical errors in our manuscript. Your observation is crucial for maintaining the high standard of scientific and academic writing. In response to your valuable input, we have conducted a thorough proofreading of our document. Our goal is to ensure a smooth and error-free reading experience for the audience.

We appreciate your diligence in reviewing our work, and we hope that the revisions address the grammatical issues you identified. Your feedback is highly valued, and we are committed to delivering a polished and refined manuscript.

References

Abbas, M., Raja, U., Darr, W., & Bouckenooghe, D. (2014). Combined effects of perceived politics and psychological capital on job satisfaction, turnover intentions, and performance. Journal of management, 40(7), 1813-1830.

Abbas, M., & Raja, U. (2015). Impact of psychological capital on innovative performance and job stress. Canadian Journal of Administrative Sciences/Revue Canadienne des Sciences de l'Administration, 32(2), 128-138.

Abbas, A., Saud, M., Suhariadi, F., Usman, I., & Ekowati, D. (2022). Positive leadership psychology: Authentic and servant leadership in higher education in Pakistan. Current Psychology, 41(9), 5859-5871.

Al Halbusi, H., Ruiz-Palomino, P., & Williams, K. A. (2023). Ethical leadership, subordinates’ moral identity and self-control: Two-and three-way interaction effect on subordinates’ ethical behavior. Journal of Business Research, 165, 114044.

Dey, M., Bhattacharjee, S., Mahmood, M., Uddin, M. A., & Biswas, S. R. (2022). Ethical leadership for better sustainable performance: Role of employee values, behavior and ethical climate. Journal of Cleaner Production, 337, 130527.

Haq, I. U., Raja, U., Alam, I., De Clercq, D., & Saleem, S. (2022). Servant leadership and mistreatment at the workplace: mediation of trust and moderation of ethical climate. Personnel Review, 51(3), 1061-1084.

Kuenzi, M., Mayer, D. M., & Greenbaum, R. L. (2020). Creating an ethical organizational environment: The relationship between ethical leadership, ethical organizational climate, and unethical behavior. Personnel Psychology, 73(1), 43-71.

Murtaza, G., Abbas, M., Raja, U., Roques, O., Khalid, A., & Mushtaq, R. (2016). Impact of Islamic work ethics on organizational citizenship behaviors and knowledge-sharing behaviors. Journal of business ethics, 133, 325-333.

Park, J. G., Zhu, W., Kwon, B., & Bang, H. (2023). Ethical leadership and follower unethical pro-organizational behavior: examining the dual influence mechanisms of affective and continuance commitments. The International Journal of Human Resource Management, 1-31.

Tang, Y., & Li, Y. (2022). Ethicality neutralization and amplification: a multilevel study of ethical leadership and unethical pro-organizational behavior. Journal of Managerial Psychology, 37(2), 111-124.
