# Supplementary material for: Unpacking the paradoxical impact of ethical leadership on employees’ pro-social rule-breaking behavior: The interplay of employees’ psychological capital and moral identity
Source: PLoS One. 2024 Aug 22;19(8):e0306912. doi: 10.1371/journal.pone.0306912 (PMC11341026; doi:10.1371/journal.pone.0306912)
Supplement: Attachment — Submitted filename: Response to Reviewers.docx [file pone.0306912.s003.docx]

**Response to Reviewers**

**Journal Requirements**

Please review your reference list to ensure that it is complete and correct. If you have cited papers that have been retracted, please include the rationale for doing so in the manuscript text, or remove these references and replace them with relevant current references. Any changes to the reference list should be mentioned in the rebuttal letter that accompanies your revised manuscript. If you need to cite a retracted article, indicate the article’s retracted status in the References list and also include a citation and full reference for the retraction notice.

**Authors’ Response**

We sincerely appreciate your prompt response and valuable guidance regarding the submission of our manuscript.

In alignment with your stipulations, we have diligently scrutinized our reference list to ensure its comprehensiveness and accuracy. Each citation in our manuscript has been meticulously examined to ascertain the absence of any references to retracted papers. Notably, our thorough review revealed no instances of such citations.

Nonetheless, we have made modifications to the revised reference list as detailed in the rebuttal letter. These changes were implemented either in response to Reviewer #3's comments on the revised literature review or to eliminate duplicate references. The updated reference list has been substituted at the end of the manuscript.

We are committed to upholding the highest standards of integrity and accuracy in our research, and we appreciate the opportunity to address this matter. Please find the revised manuscript and accompanying rebuttal letter attached for your review.

Thank you for your attention to this matter, and we look forward to your feedback.

**Reviewers' Comments**

**Reviewer # 3**

**Methodological Flaws:**

The research design and methodology employed in the study lack clarity and robustness. The absence of a clear experimental design compromises the validity of the findings. Addressing these methodological flaws is crucial for the credibility of the research.

**Authors’ Response**

We appreciate your thoughtful review of our manuscript and the opportunity to address your concerns regarding the research design and methodology employed in our study.

We acknowledge that our study did not utilize a traditional experimental design. Instead, we opted for a cross-sectional survey approach, supplemented by a time-lagged data collection method (Aguinis et al., 2021). This approach allowed us to gather data from a diverse range of hospitals and nursing staff across multiple cities, thereby enhancing the generalizability of our findings within the healthcare industry of Pakistan.

We employed purposive sampling technique, a non-probability sampling method, for data collection due to the absence of a sampling frame (Sekaran & Bougie, 2016). Despite the inherent limitations, such as potential sampling bias, associated with this method, we implemented measures to address these concerns. Notably, our sampling strategy ensured representation from both public and private hospitals in capital and provincial cities, thus encapsulating a broad spectrum of perspectives within the healthcare sector. Additionally, the adoption of a time-lagged cross-sectional design facilitated data collection from a heterogeneous sample of hospitals and nursing staff, thereby minimizing potential sampling errors (Saunders et al., 2009). Moreover, through higher sample size (515), the study sought to achieve robust population representation, mitigate potential sampling errors, and augment statistical power for the detection of significant relationships among variables.

We also note that the use of a time-lagged design helped reduce the risk of common method bias, as data were collected at three distinct time points with intervals of six to eight weeks between each phase (Podsakoff et al., 2012). This approach enabled us to examine the stability of relationships over time and to establish the temporal precedence of variables, enhancing the robustness of our findings. To counteract any effects of social desirability bias, the data collection process was meticulously designed to incorporate essential precautions suggested by the management scholars (Larson, 2019).

Although our chosen methodology may differ from traditional experimental designs, we believe it was well-suited to address the complexities of our research questions and the practical realities of conducting research in the healthcare industry of Pakistan. This approach is also supported by a number of studies exploring similar topics (Dey et al., 2022; Hsieh et al., 2020; Miao et al., 2020; Park et al., 2023; Tang & Li, 2022; Wang & Shi, 2021).

Aguinis, H., Hill, N. S., & Bailey, J. R. (2021). Best practices in data collection and preparation: Recommendations for reviewers, editors, and authors. Organizational Research Methods, 24(4), 678-693.

Dey, M., Bhattacharjee, S., Mahmood, M., Uddin, M. A., & Biswas, S. R. (2022). Ethical leadership for better sustainable performance: Role of employee values, behavior and ethical climate. Journal of Cleaner Production, 337, 130527.

Hsieh, H. H., Hsu, H. H., Kao, K. Y., & Wang, C. C. (2020). Ethical leadership and employee unethical pro-organizational behavior: a moderated mediation model of moral disengagement and coworker ethical behavior. Leadership & Organization Development Journal, 41(6), 799-812.

Larson, R. B. (2019). Controlling social desirability bias. International Journal of Market Research, 61(5), 534-547.

Park, J. G., Zhu, W., Kwon, B., & Bang, H. (2023). Ethical leadership and follower unethical pro-organizational behavior: examining the dual influence mechanisms of affective and continuance commitments. The International Journal of Human Resource Management, 1-31.

Podsakoff, P. M., MacKenzie, S. B., & Podsakoff, N. P. (2012). Sources of method bias in social science research and recommendations on how to control it. Annual review of psychology, 63, 539-569.

Miao, Q., Eva, N., Newman, A., Nielsen, I., & Herbert, K. (2020). Ethical leadership and unethical pro‐organisational behaviour: The mediating mechanism of reflective moral attentiveness. *Applied Psychology*, *69*(3), 834-853.

Saunders, M., Lewis, P., & Thornhill, A. (2009). Research methods for business students. Pearson education.

Sekaran, Uma, and Roger Bougie. Research methods for business: A skill building approach. john wiley & sons, 2016.

Tang, Y., & Li, Y. (2022). Ethicality neutralization and amplification: a multilevel study of ethical leadership and unethical pro-organizational behavior. *Journal of Managerial Psychology*, *37*(2), 111-124.

Wang, F., & Shi, W. (2021). Inclusive leadership and pro-social rule breaking: the role of psychological safety, leadership identification and leader-member exchange. Psychological Reports, 124(5), 2155-2179.

**Inadequate Literature Review:**

The literature review is insufficient in providing a comprehensive understanding of the existing research in the field. A more thorough review of relevant literature is needed to place the current study in context and demonstrate its contribution to the field.

**Authors’ Response**

Thank you for your valuable feedback. In response to your comments, we have revised the literature review section to ensure a more comprehensive coverage of relevant literature in the field. We have incorporated additional studies to provide a deeper understanding of the research landscape and the context of our study. We are confident that these revisions strengthen the manuscript and effectively demonstrate the significance and contribution of our research to the existing body of knowledge. The literature review has been revised to incorporate additional studies and the study context highlighted in green for easy reference.

**Data Analysis and Interpretation Issues**

The data analysis and interpretation in the manuscript are not sufficiently rigorous. The statistical methods employed are not well justified, and the interpretation of results lacks depth. A more robust statistical analysis and a careful interpretation of findings are essential for the paper to be considered for publication.

**Authors’ Response**

Thank you for your feedback on our manuscript and for highlighting the importance of rigor in data analysis and interpretation. We have carefully reviewed your comments and have already taken them into consideration in our manuscript.

Regarding the statistical methods employed in our study, we utilized both SPSS and PLS-SEM with Smart PLS 4 software for data analysis. SPSS was utilized for data entry, coding, and initial descriptive analysis, while PLS-SEM was employed to establish the measurement and structural models and test the proposed hypotheses. We selected these methods based on their suitability for handling the complexity of our research questions and dataset as suggested by the management scholars (Becker et al., 2023; Hair et al., 2019; Sharma et al., 2023).

To address concerns about the adequacy of our statistical analysis, we conducted various tests to evaluate the reliability, validity, and predictive relevance of our measurement and structural models. These tests included assessments of internal consistency, convergent validity, discriminant validity, collinearity, coefficient of determination (R2), effect size (F2), predictive relevance (Q2), and statistical significance of path coefficients in the light of recommendations in the SEM PLS literature (Hair et al., 2019; Legate et al., 2023; Ringle et al., 2020).

We acknowledge the importance of providing a thorough interpretation of our findings. In our manuscript, we presented detailed explanations of the results, including the significance and implications of each hypothesis test. We discussed the direction and magnitude of relationships in our analysis (Becker et al., 2023; Hair et al., 2019; Ringle et al., 2020). Moreover, we also deliberated upon the practical implications of our findings for theory and practice in the field (Please refer to the Discussion Section, Theoretical Contributions and Managerial Implications of the manuscript).

We appreciate your valuable feedback and are committed to addressing your concerns to improve the quality and rigor of our manuscript.

Becker, J. M., Cheah, J. H., Gholamzade, R., Ringle, C. M., & Sarstedt, M. (2023). PLS-SEM’s most wanted guidance. International Journal of Contemporary Hospitality Management, 35(1), 321-346.

Hair, J. F., Risher, J. J., Sarstedt, M., & Ringle, C. M. (2019). When to use and how to report the results of PLS-SEM. European business review, 31(1), 2-24.

Legate, A. E., Hair Jr, J. F., Chretien, J. L., & Risher, J. J. (2023). PLS‐SEM: Prediction‐oriented solutions for HRD researchers. Human Resource Development Quarterly, 34(1), 91-109.

Ringle, C. M., Sarstedt, M., Mitchell, R., & Gudergan, S. P. (2020). Partial least squares structural equation modeling in HRM research. The International Journal of Human Resource Management, 31(12), 1617-1643.

Sharma, P. N., Liengaard, B. D., Sarstedt, M., Hair, J. F., & Ringle, C. M. (2023). Extraordinary claims require extraordinary evidence: A comment on “Recent Developments in PLS”. Communications of the Association for Information Systems, 52(1), 739-742.

**Weak Contribution to the Field:**

The manuscript does not sufficiently contribute to the existing body of knowledge in the field. The novelty and significance of the findings are not well established, and the paper fails to make a meaningful impact on the current understanding of [topic].
**Authors’ Response**

Thank you for your feedback on our manuscript. We appreciate the opportunity to address your concerns and clarify the significance and novelty of our research in the field of ethical leadership (EL).

Our study aims to contribute to the existing body of knowledge by addressing several research gaps identified in the literature. Firstly, while previous research has extensively explored the influence of various leadership styles on employees' behaviors, there is a notable gap in understanding how EL specifically affects PSRB in organizations. By investigating this relationship, particularly in non-Western cultural contexts, our study expands our understanding of EL's effects and its implications for diverse organizations.

Secondly, we delve into the explanatory mechanism of employees' psychological capital (PsyCap) in the EL-PSRB relationship. Despite the recognition of PsyCap as a pivotal psychological resource, its role as a mediator in this context remains largely unexplored. Our study aims to fill this gap by offering insights into how EL influences employees' psychological and cognitive processes, thereby affecting their engagement in PSRB.

Furthermore, we explore the moderating role of employees' moral identity (MID) in the connection between EL and PsyCap. This investigation sheds light on the boundary conditions of EL's effectiveness, providing a more comprehensive understanding of how leadership influences employees within organizations.

Lastly, our study evaluates the applicability of social cognitive theory (SCT) in understanding workplace behaviors, particularly in the context of EL. By applying SCT to examine the intricate connection between EL and PSRB, we contribute to a deeper understanding of the cognitive and contextual factors that shape employee conduct in organizations.

Overall, our research fills significant gaps in the literature by examining the influence of EL on PSRB, elucidating the mediating mechanism of PsyCap and moderating mechanism of MID, and applying a theoretical framework of SCT to enhance our understanding of workplace behaviors. We believe that these contributions are meaningful and offer valuable insights for scholars and practitioners in the field of management and organizational behavior.

**Writing Quality:**

The overall writing quality of the manuscript needs improvement. There are numerous grammatical errors, awkward phrasing, and inconsistencies throughout the text. A thorough revision of language and style is necessary to enhance the readability of the manuscript.

**Authors’ Response**

Thank you for your feedback on the manuscript. We appreciate your thorough review and recognize the importance of enhancing the writing quality to improve the readability of the paper.

We acknowledge the need for a thorough revision of language and style to address grammatical errors, awkward phrasing, and inconsistencies in the text. To ensure clarity and coherence, we undertook the following steps shown in green throughout the manuscript:

Comprehensive Editing: We conducted a detailed review of the manuscript to identify and correct grammatical errors, including punctuation, tense consistency, and sentence structure.

Clarity and Consistency: We paid particular attention to ensure that the language used is clear and concise, eliminating any ambiguities or confusing phrases. Additionally, we ensured consistency in terminology and writing style throughout the manuscript.

Flow and Cohesion: We reviewed the organization of the text to enhance the flow of ideas and improve the overall coherence of the manuscript. This involved restructuring paragraphs, as necessary, to ensure logical progression and coherence between sections.

Proofreading: Finally, we conducted a thorough proofreading of the revised manuscript to catch any remaining errors and ensure that the text meets the journal's standards for language and style.

We are committed to addressing these concerns promptly to enhance the quality and readability of the manuscript. Thank you for providing us with the opportunity to improve the manuscript, and we look forward to submitting the revised version for your review.

**Reviewer #5:**

I am happy that authors have made significant revisions and therefore I am accepting this version. Thank you

**Authors’ Response**

We are grateful for recognizing the substantial revisions made to the manuscript. We appreciate the acceptance of this version. Your positive feedback encourages us and validates our commitment to enhancing the quality of our work. Thank you for your time and consideration throughout the review process.

**Changes to the Reference List**

Added

The following references have been incorporated into the reference list following the revision of the literature review:

[46] Tu, C. K., & Luo, B. (2020). Paternalistic leadership and pro-social rule breaking: The moderating roles of psychological empowerment and leader-member exchange. *Human Systems Management*, *39*(1), 93-103.

[52] Hattie, J., Hodis, F. A., & Kang, S. H. (2020). Theories of motivation: Integration and ways forward. Contemporary Educational Psychology, 61, 101865.

[53] Schunk, D. H., & DiBenedetto, M. K. (2021). Self-Regulation, self-efficacy, and learning disabilities. Learning Disabilities-Neurobiology, Assessment, Clinical Features and Treatments.

Deleted

The following references have been removed to eliminate redundancy from the reference list:

[48] Egorov, M., Kalshoven, K., Pircher Verdorfer, A., & Peus, C. (2020). It’sa match: Moralization and the effects of moral foundations congruence on ethical and unethical leadership perception. Journal of Business Ethics, 167, 707-723.

[61] Goswami, A. K., & Agrawal, R. K. (2023). It’sa knowledge centric world! Does ethical leadership promote knowledge sharing and knowledge creation? Psychological capital as mediator and shared goals as moderator. Journal of Knowledge Management, 27(3), 584-612.
