# Supplementary material for: Unpacking the paradoxical impact of ethical leadership on employees’ pro-social rule-breaking behavior: The interplay of employees’ psychological capital and moral identity
Source: PLoS One. 2024 Aug 22;19(8):e0306912. doi: 10.1371/journal.pone.0306912 (PMC11341026; doi:10.1371/journal.pone.0306912)
Supplement: Attachment — Submitted filename: Response to Reviewers.docx [file pone.0306912.s004.docx]

Dear Dr. Kashif Ali,

Thank you for providing us with the opportunity to revise and improve our manuscript titled "Unpacking the Paradoxical Impact of Ethical Leadership on Employees’ Pro-Social Rule-Breaking Behavior: The Interplay of Employees’ Psychological Capital and Moral Identity" (PONE-D-23-30966R2).

We have carefully considered and addressed all the comments and suggestions raised by the reviewers and the Academic Editor. Please find the following documents included with our resubmission:

1. A detailed rebuttal letter responding to each point raised by the reviewers and the Academic Editor, labeled 'Response to Reviewers'.

2. A marked-up copy of our manuscript highlighting the changes made to the original version, labeled 'Revised Manuscript with Track Changes'.

3. An unmarked version of the revised manuscript without tracked changes, labeled 'Manuscript'. A Title Page has also been included with the Manuscript as required by Solna Carreon Santos through email dated 24 June 2024.

We confirm that no changes have been made to our financial disclosures. Additionally, we have followed the guidelines for resubmitting our figure files as outlined in your previous correspondence.

We appreciate the constructive feedback and believe that the revisions have significantly enhanced the quality and clarity of our manuscript. We hope that the revised version meets the expectations of PLOS ONE and look forward to your favorable consideration.

Thank you once again for your guidance and the opportunity to resubmit our work.

Kind Regards,

Mushtaq Ahmed

Corresponding Author

Journal Requirements

Please review your reference list to ensure that it is complete and correct. If you have cited papers that have been retracted, please include the rationale for doing so in the manuscript text, or remove these references and replace them with relevant current references. Any changes to the reference list should be mentioned in the rebuttal letter that accompanies your revised manuscript. If you need to cite a retracted article, indicate the article’s retracted status in the References list and also include a citation and full reference for the retraction notice.

Authors’ Response

Thank you for your detailed guidance regarding our reference list. We have thoroughly reviewed our reference list to ensure its completeness and accuracy. To the best of our knowledge, none of the cited papers have been retracted.

We have made one update to our references: the citation by Podsakoff et al. (2012) in the Methods Section has been replaced with the more recent citation of Podsakoff et al. (2024). This change has been reflected in the reference list shown in green color.

We appreciate the opportunity to revise our manuscript and hope that the updated references meet the journal's requirements.

Response to Reviewers

Reviewer # 3

Reviewer’s Comment

The hypotheses proposed in the study lack clarity and specificity. It is challenging to discern the precise relationships between ethical leadership, employees' psychological capital, moral identity, and pro-social rule-breaking behavior. Without clear hypotheses, the study's theoretical framework and research objectives remain ambiguous.

Authors’ Response

Thank you for your valuable feedback on the proposed hypotheses of our study. We would like to clarify that our hypotheses have been formulated in line with recent literature in management sciences (Ahmed & Khan, 2023; Hsieh et al., 2020; Miao et al., 2020; Park et al., 2023; Tang & Li, 2022). In response to your comments, we have further revised the literature review section to enhance the clarity and specificity of our hypotheses. These revisions, highlighted in green, can be found on pages 6 to 13. We believe these changes address the concerns raised and significantly improve the quality and coherence of our manuscript. We appreciate the opportunity to enhance our work.

Reviewer’s Comment

The introduction needs to be focused on relevant literature, currently, it seems verbose and difficult to follow.

Authors’ Response

Thank you for your insightful feedback on the Introduction of our manuscript. We understand the importance of a concise and focused introduction. In our previous revisions, we expanded the introduction to address other reviewers’ comments regarding the research gaps related to ethical leadership (EL), employees’ pro-social rule-breaking behavior (PSRB), the mediating role of psychological capital (PsyCap), and the moderating role of moral identity (MID). This expansion was necessary to ensure clarity and specificity in response to those comments.

However, in light of your feedback, we have further refined our introduction to enhance its focus on relevant literature and improve readability. The revised introduction is now more concise, clearly contextualizing our study. The changes have been made on Pages 2-3 and are highlighted in green. Thank you for your understanding and consideration.

Reviewer’s Comment

The sampling has to be explained and justified in a manner that could be replicated in other places and defined well.

Authors’ Response

We appreciate the reviewer's thoughtful consideration of our sampling methodology and its potential for replication in varied contexts. Our study employed a purposive sampling strategy to select registered nursing staff from a diverse array of public and private hospitals across major cities in Pakistan. This methodological choice was necessitated by the absence of a comprehensive sampling frame covering all healthcare institutions nationwide, a common challenge in studies conducted within complex organizational environments such as healthcare.

The decision to include 515 participants was guided by established guidelines for structural equation modeling (SEM) studies, ensuring robust statistical power to detect significant relationships among variables. This sample size aligns with recommended practices in the methodological literature pertaining to SEM research (Comrey & Lee, 2013; Jobst et al., 2023; Lakens, 2022; Memon et al., 2020).

Furthermore, our data collection procedures were meticulously designed to mitigate potential biases. We employed a time-lagged cross-sectional research design spanning three distinct phases, each separated by eight-week intervals. This approach not only minimized common method biases but also facilitated the examination of temporal relationships among EL, PsyCap, MID and PSRB (Aguinis et al., 2021; Haider et al., 2019; Podsakoff et al., 2024).

Our engagement with hospital management was formalized through university authorization letters, securing official approval and logistical support from administrative authorities. This approach ensured compliance with ethical standards and institutional guidelines governing research involving human participants.

Overall, the sampling methodology detailed extensively on Pages 14-16 shown in green was crafted with careful consideration of practical constraints and methodological rigor. We believe these details elucidate the rationale behind our approach and underscore its potential for replication in diverse healthcare settings (Aguinis et al., 2023; Podsakoff et al., 2024). We are grateful for the reviewer’s insightful feedback, which has prompted us to clarify these methodological strengths.

Reviewer’s Comment

The study's theoretical contribution is unclear. While the interplay between ethical leadership, psychological capital, moral identity, and pro-social rule-breaking behavior is an interesting topic, the study fails to provide novel insights or advance existing theoretical frameworks. Theoretical implications should be clearly articulated and supported by the empirical findings.

Authors’ Response

We appreciate your comments on the theoretical contributions of our study. It is highlighted that the theoretical contributions of our study have been clearly articulated throughout the manuscript. In the Introduction highlighted in green color (Pages 4-5), we delineated four key research objectives that address significant gaps in understanding the interplay between EL, PsyCap, MID, and PSRB. These objectives collectively enrich existing theoretical frameworks by exploring EL's influence in non-Western contexts, elucidating PsyCap as a mediator, and highlighting MID as a moderator in the EL-PsyCap relationship.

In the Discussion highlighted in green color (Pages 33-36) and Theoretical Contributions highlighted in green color (Pages 36-38), we substantiated these theoretical implications with empirical findings. Notably, our study revealed an unexpected positive association between EL and PSRB in the healthcare context, challenging traditional assumptions and underscoring the contextual nuances influencing leadership effects. Moreover, by demonstrating PsyCap's mediating role and MID's moderating influence, we extend Social Cognitive Theory's (SCT) applicability, offering insights into how individual differences shape leadership outcomes.

These findings collectively contribute to a nuanced understanding of leadership dynamics, emphasizing the role of cognitive processes, individual differences, and organizational contexts in shaping workplace behaviors. We appreciate the reviewer's feedback, which has prompted us to underscore these theoretical contributions more explicitly.

Reviewer’s Comment:

The methodological approach employed in the study raises concerns about its validity and reliability. The use of self-report measures for variables such as ethical leadership, psychological capital, moral identity, and pro-social rule-breaking behavior may introduce common method bias and social desirability bias, thereby compromising the validity of the results. Additionally, the cross-sectional design limits the ability to draw causal inferences and establish temporal precedence.

Authors’ Response:

Thank you for your insightful feedback regarding the validity and reliability of the methodological approach employed in our study. We appreciate your concerns and have taken the opportunity to address them comprehensively.

We understand your concerns regarding the use of self-report measures for variables such as EL, PsyCap, MID, and PSRB. We would like to provide a detailed explanation of our rationale for using these measures and the steps we took to mitigate potential biases.

The constructs under investigation—EL, PsyCap, MID, and PSRB—are inherently subjective and best captured through employees' perceptions. Self-report measures are widely accepted in organizational research for assessing personal attitudes, beliefs, and behaviors, as noted by Kobe et al. (2001). For instance, ethical leadership pertains to how employees perceive their leaders' ethical conduct, which can only be accurately reported by the employees themselves. The use of self-report measures for these constructs is grounded in established research practices, with recent studies in the field successfully employing similar instruments (Ahmed & Khan, 2023; Hsieh et al., 2020; Miao et al., 2020; Park et al., 2023; Tang & Li, 2022).

To address concerns about common method bias, we implemented a time-lagged cross-sectional design, collecting data in three distinct phases over an eight-week period (Haider et al., 2019). This approach reduces the likelihood of common method variance, consistent with the recommendations by Podsakoff et al. (2024). Additionally, to counteract social desirability bias, we ensured strict confidentiality and anonymity of responses. Participants were assured that their inputs would remain confidential, and no identifying information was collected, fostering an environment where employees felt safe to provide honest and accurate responses, as highlighted by Larson (2019).

The measurement scales used in our study were validated through reliability analysis (Becker et al., 2023; Hair et al., 2019). The Cronbach's alpha coefficients for the scales ranged from 0.915 to 0.937, indicating high internal consistency reliability. This rigorous validation process helps to ensure that the self-report measures accurately capture the constructs of interest. While we acknowledge the limitations of a cross-sectional design in establishing causality, our time-lagged approach allows for inferences regarding temporal relationships among the variables (Podsakoff et al., 2024). This design provides valuable insights into the dynamics of ethical leadership, psychological capital, moral identity, and pro-social rule-breaking behavior over time within the healthcare context.

Our sample size of 515 participants, determined based on statistical guidelines and prior research, enhances the robustness and generalizability of our findings. This large sample size bolsters the statistical power of our study and reinforces the validity of our conclusions (Comrey & Lee, 2013; Jobst et al., 2023; Lakens, 2022; Memon et al., 2020). Therefore, we believe that our methodological choices, including the use of self-report measures, were carefully designed to ensure the validity and reliability of our findings (Aguinis et al., 2023). The steps taken to mitigate potential biases and the robust sample size strengthen the credibility of our study.

We thank the reviewer for prompting us to clarify these aspects further.

Reviewer’s Comment

The discussion of findings is superficial and fails to critically engage with the results. The authors merely summarize the findings without offering meaningful interpretations or insights. A thorough discussion of the implications of the results for theory, research, and practice is essential for demonstrating the study's significance.

Authors’ Response

We appreciate the reviewer's feedback and have carefully revisited our Discussion Section shown in green color (Pages 33 – 36), Theoretical Contributions (Pages 36 – 38), and Managerial Implications (Pages 38 – 39) to address this concern. In these revised sections, we have focused on providing deeper insights and interpretations of our findings, particularly in relation to their theoretical and practical implications. We have critically examined the unexpected positive association between EL and PSRB in the healthcare context, challenging conventional wisdom and highlighting contextual factors influencing leadership dynamics. Furthermore, we have elucidated how PsyCap mediates this relationship, shedding light on the underlying mechanisms through which EL influences employee behaviors.

The Theoretical Contributions section shown in green color (Pages 36 – 38,) now underscores the study's contributions to existing theory, emphasizing the expansion of SCT by integrating contextual factors and individual differences. We discuss how these findings enrich our understanding of leadership effects in non-Western settings, providing nuanced insights that contribute to broader theoretical frameworks in organizational behavior.

Additionally, the Managerial Implications section shown in green color (Pages 38 – 39) has been enhanced to outline practical recommendations based on our findings. We emphasize the importance of fostering ethical leadership practices in healthcare organizations, considering the implications for organizational culture and employee well-being. These insights are intended to guide leaders and practitioners in implementing effective leadership strategies that promote ethical behavior and enhance organizational effectiveness.

Overall, we believe these revisions strengthen our discussion by offering a comprehensive analysis of the implications of our results for theory, research, and practice. We thank the reviewer for prompting us to enrich these sections with deeper critical engagement and meaningful interpretations.

References

Aguinis, H., Bergh, D., & Molina-Azorin, J. F. (2023). Methodological challenges and insights for future international business research. Journal of International Business Studies, 54(2), 219-232.

Aguinis, H., Hill, N.S. and Bailey, J.R., 2021. Best practices in data collection and preparation: Recommendations for reviewers, editors, and authors. Organizational Research Methods, 24(4), pp.678-693.

Ahmed, M., & Khan, M. I. (2023). Beyond the universal perception: Unveiling the paradoxical impact of ethical leadership on employees’ unethical pro-organizational behavior. Heliyon, 9(11).

Becker, J. M., Cheah, J. H., Gholamzade, R., Ringle, C. M., & Sarstedt, M. (2023). PLS-SEM’s most wanted guidance. International Journal of Contemporary Hospitality Management, 35(1), 321-346.

Comrey, A. L., & Lee, H. B. (2013). A first course in factor analysis. Psychology press.

Haider, S., de Pablos Heredero, C. and Ahmed, M., 2019. A three-wave time-lagged study of mediation between positive feedback and organizational citizenship behavior: the role of organization-based self-esteem. Psychology research and behavior management, pp.241-253.

Hair, J. F., Risher, J. J., Sarstedt, M., & Ringle, C. M. (2019). When to use and how to report the results of PLS-SEM. European business review, 31(1), 2-24.

Hsieh, H. H., Hsu, H. H., Kao, K. Y., & Wang, C. C. (2020). Ethical leadership and employee unethical pro-organizational behavior: a moderated mediation model of moral disengagement and coworker ethical behavior. Leadership & Organization Development Journal, 41(6), 799-812.

Jobst, L. J., Bader, M., & Moshagen, M. (2023). A tutorial on assessing statistical power and determining sample size for structural equation models. Psychological Methods, 28(1), 207.

Kobe, L. M., Reiter-Palmon, R., & Rickers, J. D. (2001). Self-reported leadership experiences in relation to inventoried social and emotional intelligence. Current Psychology, 20, 154-163.

Lakens, D. (2022). Sample size justification. Collabra: Psychology, 8(1), 33267.

Larson, R. B. (2019). Controlling social desirability bias. International Journal of Market Research, 61(5), 534-547.

Memon, M. A., Ting, H., Cheah, J. H., Thurasamy, R., Chuah, F., & Cham, T. H. (2020). Sample size for survey research: Review and recommendations. Journal of Applied Structural Equation Modeling, 4(2), 1-20.

Miao, Q., Eva, N., Newman, A., Nielsen, I., & Herbert, K. (2020). Ethical leadership and unethical pro‐organisational behaviour: The mediating mechanism of reflective moral attentiveness. Applied Psychology, 69(3), 834-853.

Park, J. G., Zhu, W., Kwon, B., & Bang, H. (2023). Ethical leadership and follower unethical pro-organizational behavior: examining the dual influence mechanisms of affective and continuance commitments. The International Journal of Human Resource Management, 34(22), 4313-4343.

Podsakoff, P. M., Podsakoff, N. P., Williams, L. J., Huang, C., & Yang, J. (2024). Common method bias: it's bad, it's complex, it's widespread, and it's not easy to fix. Annual Review of Organizational Psychology and Organizational Behavior, 11, 17-61.

Tang, Y., & Li, Y. (2022). Ethicality neutralization and amplification: a multilevel study of ethical leadership and unethical pro-organizational behavior. Journal of Managerial Psychology, 37(2), 111-124.

Reviewer # 6

Reviewer’s Comment

Authors have incorporated all changes/suggestions. However, it is suggested to reconsider the title of article

Author’s Response

Thank you for your valuable feedback. We appreciate your suggestion regarding the title of our article. After careful consideration of the topic and its objectives, we believe that the current title aligns well with the aims of our study. We are grateful for the reviewer’s continued guidance in enhancing the quality of our manuscript.
